# Editorial: Nanocellulose: A Multipurpose Advanced Functional Material, Volume II

**DOI:** 10.3389/fbioe.2022.931256

**Published:** 2022-05-19

**Authors:** Muhammad Wajid Ullah, Mazhar Ul-Islam, Fazli Wahid, Guang Yang

**Affiliations:** ^1^ School of the Environment and Safety Engineering, Biofuels Institute, Jiangsu University, Zhenjiang, China; ^2^ Department of Chemical Engineering, Dhofar University, Salalah, Oman; ^3^ Department of Biomedical Sciences, Pak-Austria Fachhochschule: Institute of Applied Sciences and Technology, Haripur, Pakistan; ^4^ Department of Biomedical Engineering, College of Life Science and Technology, Huazhong University of Science and Technology, Wuhan, China

**Keywords:** nanocellulose, yield, modification, composites, hydrogels, environment, textile, tissue engineering

According to the morphology and source, there are three classes of nano-scale cellulose (i.e., nanocellulose), including cellulose nanocrystals (CNCs), cellulose nanofibers (CNFs), and bacterial nanocellulose (BNC), where the first two types are obtained from plants (Yadav et al., 2021) while the last one is obtained from the microbial origin (Ullah et al., 2017). Besides, nanocellulose is obtained from algae (Ruan et al., 2018) and animals (Bacakova et al., 2019) as well as synthesized by the cell-free enzyme systems (Kim et al., 2019).

Presently, nanocellulose research is conducted from three main aspects: production, quality enhancement, and functionalization for various biotechnological applications. For instance, plant-derived cellulose contains lignin, hemicellulose, and minerals, which should be removed to obtain nanocellulose of high purity and quality (Ul-Islam et al., 2019a). To this end, efforts have been carried out to develop green approaches to minimize or bypass the use of toxic chemicals required for the hydrolysis of lignocellulosic materials. On the other hand, BNC production by bacteria suffers from low yield and productivity and a high production cost. Therefore, several strategies such as strain improvement, co-culturing, and development of engineered strains and advanced reactors have been adopted to enhance the yield and productivity of BNC (Islam et al., 2017; Sajadi et al., 2019; Moradi et al., 2021). At the same time, different agro-industrial wastes have been utilized as the carbon sources for BNC production by bacteria (Velásquez-Riaño and Bojacá, 2017; Ul-Islam et al., 2020; Zhou et al., 2021). Similarly, although different types of nanocellulose possess impressive morphological and physico-chemical properties and are non-toxic, these do not possess some desired properties of materials like adhesive sites, antimicrobial and antioxidant activities, electromagnetic properties, and catalytic activity, and thus require further modification (Picheth et al., 2017; Vilela et al., 2019). Due to similar surface chemistry, all types of nanocellulose are modified by somehow same chemical strategies like esterification (Spinella et al., 2016), etherification (De La Motte et al., 2011), amidation (Kim et al., 2015), and oxidation (Khattak et al., 2021) as well as modified physically through hydrogen bonding, electrostatic interaction, hydrophilic/hydrophobic interaction, and *π*-π stacking, where the free OH groups of cellulose directly interact with an electron-rich amine group, oxygen atom, and carboxyl group and form hydrogen bond (Ullah et al., 2019). Due to the unique surface chemistry, diversity, and impressive features of different types of nanocellulose, these find applications in areas like biomedical (Wang et al., 2021), environment (Shoukat et al., 2019), textile (Felgueiras et al., 2021), pharmaceutics (Raghav et al., 2021), energy (Zhang et al., 2020), additive manufacturing (Fourmann et al., 2021), cosmetics (Bianchet et al., 2020), bioelectronics (Khan et al., 2015), food (Atta et al., 2021; Haghighi et al., 2021), and others. Herein, the plant-derived cellulose is preferably used in the development of low-cost materials for environmental, packaging, and other applications, while BNC is mostly utilized in the production of high value-added products for biomedical applications. However, there is no clear distinction of using any kind of cellulose for biomedical and other applications, and the debate of cost-analysis, pilot-scale production, and commercialization of nanocellulose-based products is still going on.

Considering the abundance, renewability, and interesting morphological, physiological, chemical, and biological features, this Research Topic in Frontiers in Bioengineering and Biotechnology, Sections “Biomaterials” and “Nanobiotechnology” entitled “Nanocellulose: A Multipurpose Advanced Functional Material, Volume II” is aimed to discuss the production, modification, quality enhancement, cause-effect and structure-function relationships, research trend, and multipurpose applications of different types of nanocellulose. This Research Topic contains a total of six articles, including four original research articles, one review, and one systematic review, contributed by the experts in the field.

Efforts have been devoted to enhancing the yield and productivity of BNC by utilizing active bacterial strains. To date, various acetic-acid bacteria have been evaluated for BNC production, among which *Komagataeibacter xylinus* is the most commonly used strain due to its high BNC production ability. *K. xylinus* exists in a microbial consortium called Kombucha. Wood et al. investigated the effect of repeated sub-culturing on the microbial communities and their subsequent impact on the BNC production ability of *K. xylinus* in isolated form and in Kombucha. After three cycles of sub-culturing, Kombucha produced thicker BC pellicles compared to the isolated *K. xylinus*; nevertheless, of similar nanofibrillar structures. Importantly, the highest BNC yield was obtained after the third cycle of sub-culturing. These results indicate that using Kombucha as inoculum represents a reproducible and sustainable model for high yield BNC production.

The physico-chemical modification and the development of nanocellulose-based composites with other materials, like polymers, nanomaterials, clays, and micro- and macro-molecules, not only enhance its innate features but also imparts additional functionalities. In contrast to the physical modification involving the blending of nanocellulose with a reinforcing material, the chemical modification of nanocellulose is carried out through different chemical reactions. For instance, oxidation of cellulose is carried out by treating it with 2,2,6,6-Tetramethylpiperidine-1-oxyl (TEMPO), NaBR, NaClO, etc. Suárez-Avendaño et al. carried out the TEMPO-oxidation of BNC and comparatively analyzed the mercury removal efficiency of unmodified and TEMPO-oxidized BNC from wastewater. TEMPO-oxidation altered the nanoribbon network of BNC, which in turn decreased the crystallinity of cellulose fibers. Compared to the unmodified BNC (31.6%), the TEMPO-oxidized BNC removed as high as 93% mercury from wastewater. Despite the availability of a variety of physico-chemical modification methods, nanocellulose cannot be modified with hydrophobic materials due to its hydrophilic nature. In a study, Ali et al. modified cotton-derived CNCs with 2-carboxyethyl acrylate, which improved its hydrophobic behavior as indicated by the increased contact angle measurement. The modified CNCs showed improved mechanical, thermal, and adhesive properties. The modified 2-carboxyethyl acrylate-modified CNCs were then modified with a hydrophobic E-51 epoxy resin system, which showed high shear stress, toughness, degradation, thermal stability, and recyclability. In another study, Zeng et al. carried out plasticization of microcrystalline cellulose (MCC) films by using the Chinese leak (CL, Allium tuberosum) extract. Physical mixing of MCC with CL at a 7:3 ratio showed a successful integration of lignin, polysaccharides, pectin, and waxes from CL into the MCC matrix, which acted as plasticizers to improve the mechanical strength, hydrophilicity, and UV shielding performance of the MCC/CL composite film.

According to the targeted applications, the different forms of nanocellulose are used in the form of hydrogels, aerogels, membranes, sheets, thin/thick films, fibers, emulsions, coatings, and tubes, etc. The biomedical applications of nanocellulose utilize hydrogels (Shi et al., 2016) due to their hydrophilic character, porous and fibrous structure, and maintaining a three-dimensional structure with good mechanical strength. The review of Wang et al. summarized the tissue engineering and regenerative medicine applications of CNC, CNF, and BNC-based hydrogels. The unique features of different nanocellulose-based hydrogels make them suitable biomaterials for developing tissue scaffolds (Khan et al., 2022), drug carriers (Raghav et al., 2021), 3D printed structures (McCarthy et al., 2019), wound dressing materials (Mao et al., 2021), synthetic organs (Klemm et al., 2018; Khan et al., 2021), and platforms for developing sensing (Farooq et al., 2020) and diagnostic materials (Ul-Islam et al., 2019b).

BC research has been expanding rapidly and vastly over the last couple of decades. Ho et al. carried out a bibliometric analysis to describe the trend of BNC research in the last 15 years. They selected parameters like annual outputs, citation count, key journals, countries, key academic and research institutions, research directions, and categories of the Web of Science by using keywords extracted from words in the publication title, keywords, and *KeyWords Plus*. The results showed that the number of scholarly articles on BNC increased from 2005 to 2009, with a rapid increase occurring in the last 5 years (2015–2020). The data showed that the BNC research is expanded worldwide, including in Asia, and to areas like polymer science, materials science and engineering, biomaterials science, applied chemistry, etc., and it is predominantly used in biomedical, food, environment, energy, and textile sectors. While most of the research is focused on quality enhancement (predominantly the mechanical strength) and exploring new applications, a considerable amount of effort has been devoted to decreasing its production costs, such as by utilizing low-cost substrates and different agro-industrial wastes as the carbon sources, which additionally address some of the environmental concerns of safe disposal of such wastes.

In summary, this Research Topic on nanocellulose highlights the importance of this valuable biopolymer by covering the research about its production, modification, quality enhancement, and diverse application in areas like biomedicine, environment, textile, and packaging, etc.
